# Forecasting care seekers satisfaction with telemedicine using machine learning and structural equation modeling

**DOI:** 10.1371/journal.pone.0257300

**Published:** 2021-09-24

**Authors:** Khondker Mohammad Zobair, Louis Sanzogni, Luke Houghton, Md. Zahidul Islam

**Affiliations:** 1 Department of Business Strategy and Innovation, Griffith Business School, Griffith University, Brisbane, QLD, Australia; 2 Institute for Integrated and Intelligent Systems, Griffith Sciences-Centres and Institutes, Griffith University, Brisbane, QLD, Australia; 3 Department of Computer Science and Engineering, Khulna University, Khulna, Bangladesh; Bucharest University of Economic Studies, ROMANIA

## Abstract

Many individuals visit rural telemedicine centres to obtain safe and effective health remedies for their physical and emotional illnesses. This study investigates the antecedents of patients’ satisfaction relating to telemedicine adoption in rural public hospitals settings in Bangladesh through the adaptation of Expectation Disconfirmation Theory extended by Social Cognitive Theory. This research advances a theoretically sustained prediction model forecasting patients’ satisfaction with telemedicine to enable informed decision making. A research model explores four potential antecedents: expectations, performance, disconfirmation, and enjoyment; that significantly contribute to predicting patients’ satisfaction concerning telemedicine adoption in Bangladesh. This model is validated using two-staged structural equation modeling and artificial neural network approaches. The findings demonstrate the determinants of patients’ satisfaction with telemedicine. The presented model will assist medical practitioners, academics, and information systems practitioners to develop high-quality decisions in the future application of telemedicine. Pertinent implications, limitations and future research directions are endorsed securing long-term telemedicine sustainability.

## Introduction

Telemedicine is a promising, growing beneficial approach to healing, providing remotely based support to the medically underprivileged and sparsely populated regions with inadequate access to health-based facilities. Despite the increasing demand for telemedicine, the adoption rate is below expectations [1]. Research on telemedicine initiatives demonstrates that it often fails to secure its deployment goals. Al-Samarraie, Ghazal, Alzahrani, and Moody revealed that 75% of projects are abandoned or incapable of last in Middle Eastern countries, which is as high as 90% in developing countries [2]. Within developed country’s context, Uscher-Pines et al. [3] reported that in the USA, the adoption rate is increasing < 20% of licenced treatment facilities offered telemedicine by 2019, but this rate remained lower than the adoption for telemedicine by other organisations; in 2016, 26% of all mental facilities, and 58% of all hospitals used telemedicine for various health conditions. Findings remain inconsistent, making informed judgement and conclusion about adoption issues difficult due to insufficient empirical evidence [4], particularly in sparsely populated rural communities [5].

Telemedicine growth has been concurrent with the growth of technological advancement in the 20th and 21st Centuries and sought to overcome distance in healthcare services delivery, particularly in the US and then replicated globally [6]. Telemedicine potentially improves accessibility, promotes service quality, reduces healthcare costs [7], and increases stakeholders’ choice and convenience. Many health professionals and care seekers welcome this innovative healthcare provision [7] and it’s increasingly being deployed in numerous clinical facilities [8] to sound effects on both developed and developing countries hospital settings. Telemedicine plays a pivotal role in reducing health disparity [9] between uneven health distributions in rural and urban settings, and is geared towards safeguarding health concerning steadiness.

The Bangladeshi Government has given higher priority to the integration of telemedicine into its public healthcare systems [10] to support rural and remote areas where around 70% of the population lives [11]. The formal public telemedicine healthcare systems were implemented in 2010 in rural Bangladesh [10] to make the partial fulfilment of the Digital Sonar Bangla Vision in 2021 a reality [12]. Despite the passion for public telemedicine schemes in Bangladesh, the functional adoption into clinical practices has persisted insufficient in rural settings. However, a recent study by Zobair et al. [10] highlighted the presence of 84 active telemedicine centres in 57 of Bangladesh’s 488 Upazila (i.e. subdistrict) public hospitals. The integration of 27 specialised, and district-level medical colleges and hospitals steadily providing specialist healthcare support to existing rural telemedicine centres is considered a momentous shift and one of the most significant developments in the public healthcare sector in Bangladesh [10].

Many satisfied care seekers are eventually expected to return to telemedicine for obtaining primary remedies concerning their mental and physical illness [13]. While care givers facilitate a variety of individually Information and Communication Technology (ICT) supported health services (i.e., telemedicine, telehealth, e-Health and m-health), individuals can actively participate in choosing what is more appropriate and conveniently satisfying to them [14]. For example, Anderson and Sullivan [15] reported that there is rising managerial interest in consumer satisfaction as a means of assessing service quality, which is believed to be the key indicator of organisational future reputations and growth. Hence, it is mandatory to understand consumers’ post-consumption evaluation of product/service quality, given pre-consumption expectations for forming their satisfaction judgement [15]. Literature indicates that patients’ satisfaction is a significant and influential indicator in healthcare intervention [16].

To date, past contributions concerning satisfaction with telemedicine have mostly focused on general measures of satisfaction. For example [6, 17–21], investigate antecedents to satisfaction with telemedicine without theorising or advocating novel techniques in the unique context of telemedicine. Although, a recent study by Kissi et al. [22] for instance, applied the technology acceptance model to predict physicians’ satisfaction with telemedicine. Interestingly, they point to research on the antecedents to physicians’ satisfaction with telemedicine, without validating satisfaction using Expectation Disconfirmation Theory (EDT), which remains a dominant method to study satisfaction proposed and developed by Oliver [23, 24]. Measured by the impact and amount of research conducted within satisfaction, it is fair to comment that EDT has been the dominant theory used to study satisfaction [25]. This implies that the existing satisfaction literature pertinent to telemedicine is not methodologically up to date in the way the investigations had been approached. This is supported by Whitten and Mair [26], who criticised that by digging beneath the surface of telemedicine research, the findings provide evidence that illustrates the futility of attempting to generalise satisfaction across all telemedicine frameworks. Nevertheless, telemedicine appears well accepted by rural health seekers in many developed countries [27], indicating their positive attitudes and evaluations for these services. Conversely, care seekers’ satisfaction judgments concerning telemedicine in developing countries could be based on different determinants, for example, service expectations, performance, disconfirmation, and enjoyment that actively contribute to the formation of the cognitive behaviours [28], stimulating their evaluative judgement of satisfaction decisions [29]. Following that positioning, a careful and thorough critique unique theoretical and methodological perspectives is warranted.

In contrast, Information Systems (IS) satisfaction literature [30–35] studied the antecedents to users’ satisfaction. Surprisingly, these authors have not applied EDT to explore how consumers’ expectations, performance and disconfirmation, contribute to forming their satisfaction judgements. Evidence suggests that the theoretical importance and widespread use of EDT in practice is highly important, as valid and reliable measures need to be ensured [36]. Another dominant antecedent to satisfaction is enjoyment (i.e., intrinsic motivation) from Social Cognitive theory (SCT) [37], but is consistently missing from many IS satisfaction articles. For example, a prior study by Venkatesh [38] revealed that intrinsic motivation role as a lever generating favourable user perceptions has not been adequately exploited. Subsequent empirical work by Vallerand [39] acknowledged that enjoyment and satisfaction are strongly allied.

From a methodological standpoint, some researchers theorised that satisfaction within telemedicine research had methodological and analytical limitations. For example, Kissi et al. [22] applied Technology Acceptance Model (TAM) to investigate health providers satisfaction with telemedicine, while Serrano et al. [40] applied EDT to identify patient satisfaction with telemedicine for diabetic retinopathy screenings. Surprisingly, they adopted a linear modelling approach. Venkatesh and Goyal [41] suggested that linear models are unable to reveal complexities that are expected in theories, and lead to oversimplifying the complexity of the joint effects of the components. In contrast, IS satisfaction research, for example [41, 42], applied a polynomial regression (a non-linear analysis) to better explained EDT theory. Following their work, this research applies a unique non-linear methodological approach using an Artificial Neural Network, recognizing both linear and non-linear relationships [43]. It is imperative to understand that both EDT and SCT are quite generalisable theories which have been applied to various contexts/settings. Thus, adding a new context of telemedicine may not truly add adequate contributions or values to the existing telemedicine literature, unless specific/alternative theorisations pertinent to the context of telemedicine are forwarded. Apart from the research conducted by Serrano et al. [40], scant research has been done on antecedents to patients’ satisfaction with telemedicine by employing EDT. This is surprising given this research focus on the context of customised theory building (integrated EDT and SCT), development, and testing pertinent to telemedicine. The authors anticipate that this alternative theorisation and unique methodological approach (i.e., Artificial Intelligence (AI)) will answer the crucial research questions by better grounding the phenomenon of satisfaction with telemedicine further unwrapping its contribution to uncover how antecedents to satisfaction contribute to predicting patients’ decisions towards future continuity of telemedicine.

Satisfaction incorporates individuals’ psychological and economic considerations related to product/service expectations and performance perceptions [44]. Albeit, measuring patients’ satisfaction related to telemedicine exposure is an onerous task due to interaction with numerous stakeholders (i.e., physicians, patients, nurses, and administrative and ICT staff) and technology stimuli [45]. These issues further compound the complexity of quantifying the comparability of satisfaction cognitions across all stakeholders (i.e., patients, and health providers) and the technologies (i.e., telemedicine) [44]. Hoque and Sorwar [46] for example, discovered that in Bangladesh ICT mediated healthcare services (i.e., m-Health), are investigated on the supply side (i.e., providing infrastructure), with little attention been devoted to the demand side (i.e., patients’ satisfaction). Another recent study by Anderberg et al. [47] reminded that there is a pressing need to study and predict care seekers’ behaviours towards the efficacy of telemedicine pursuits, which is becoming increasingly critical as the number of health technology solutions accelerates. Care seekers satisfaction in the present study refers to the extent of a patient’s evaluations resulting from a telemedicine service experience [48].

Evidence suggests that current healthcare services are becoming heavily reliant on technology [49]; thus, thoughtful research on predicting patients satisfaction with technology facilitated telemedicine appears critical for strengthening and sustaining this provision. Machine Learning (ML) and AI technologies have currently been gained from a wide range of special attractions for health informatics research. Shaikh et al. [50] for instance, indicated that AI particularly, Artificial Neural Networks have been used to predict future health risks. AI has been actively researched as helpful for humans [51] to transcend limitations. Vallée et al. [52] state that Artificial Neural Networks are valuable in enabling the interpretation of complex phenomenon, discovering new patterns and predicting outcomes. Artificial Neural Network (ANN) or Deep Neural Network (DNN) have seldom been used to predict patients’ satisfaction with telemedicine experience in emerging economies’ rural settings including Bangladesh.

Several classic theories have been proposed to better understand and explore the determination of consumers’ satisfaction judgements concerning products/services (i.e., Expectancy Disconfirmation Theory [24, 29, 53, 54], Assimilation Theory [29, 55–57], Equity Theory [29], Attribution Theory [29, 58], and Performance Theory [29]) in consumer satisfaction literature [29]. These satisfaction theories are rarely practised in ICT-mediated healthcare services, particularly in telemedicine research within developing countries context.

To our knowledge, no theoretically sustained prediction grounded research satisfactorily disaggregated the antecedents to care seekers’ (i.e., patients) satisfaction with telemedicine, particularly in emerging economies including Bangladesh. The existing Bangladeshi literature for instance, has not yet explored how antecedents of patients’ service continuity behaviour (i.e., expectations, performance, disconfirmation, and enjoyment) interact in forming, predicting and forecasting satisfaction with telemedicine experience. It is, therefore, imperative and essential to determine the elements (i.e., antecedents) that lead to form patients’ satisfaction, and to incorporate those antecedents are deemed crucial to predicting and forecasting future telemedicine adoptability and sustainability. To the authors’ knowledge, less emphasis has been devoted to this content in both developed and developing countries’ telemedicine research, while patients’ satisfaction experience significantly contribute to embedding their pre-consumption (i.e., expectations) and post-consumption (i.e., performance) cognitions [54] in the process leading towards their satisfaction judgement. Rarely do researchers in emerging economies reflect on this vital question. Moreover, there have been scantly reported investigations into integrated EDT and SCT and validated using a two-staged Partial Least Square Structural Equation Modeling (PLS-SEM) and DNN approach in the telemedicine domain, representing a considerable knowledge gap in the current health informatics literature. This research therefore investigates ways in which DNN can assist telemedicine to better enable predictions, increase patient recovery and satisfaction and support health professionals in better quality decision making.

## Theoretical model and hypotheses

Consumers’ products and services satisfaction have captured considerable interest [59] in various consumption settings. A growing wave of interest in research on consumer satisfaction has provoked diverse reflective interpretations of the causes and effects of satisfaction cognitions [24]. A plethora of prior research on antecedents of satisfaction, for example [23–25, 29, 53, 59], confirmed that consumers’ products/services satisfaction is prominent in the field of marketing and plays a critical role in the formation of satisfaction [60] however, satisfaction decisions are complex [23] phenomenon as related to peoples’ consumption behaviours [57]. Within the context of product evaluation and consumer satisfaction studies, R. E. Anderson proposed four theories, namely, Cognitive Dissonance Theory, Contrast Theory, Generalised Negativity Theory, and Assimilation-Contrast Theory [55]. The study acknowledged that consumerism is an interrelated complex phenomenon that incorporates ecological, social, political, ethical, economic, and technological dimensions, which need to be studied separately. Another study by LaTour and Peat [59] reminded that extensive research is desirable to fully establish the antecedents of satisfaction associated with numerous kinds of products and services/systems.

Within the marketing context, Churchill Jr and Surprenant [61] determined that satisfaction serves a significant role culminating in purchases and consumptions and post-purchase phenomenon (e.g., attitude change, repeat purchase, and brand loyalty).

In the IS literature, Venkatesh and Goyal [41], for example, stated that expectations and disconfirmation (fulfilment of expectations) contribute to the formation of satisfaction judgement. Another IS study by Bhattacherjee [62] examined how individuals’ cognitive beliefs (i.e., satisfaction/dissatisfaction judgement) influence their continuance or discontinuance behaviours. For example, individuals’ service continuance behaviour is entirely dependent on their satisfaction with the service quality and the service usefulness [62]. To understand an individual’s satisfaction is crucial because it is a vital indicator of ICT success, a key predictor of continuance intention of ICT usage/adoption [63].

### Conceptual definition of antecedents to satisfaction

Satisfaction is a complex concept that can be defined in a variety of consumption settings, including health information systems (i.e., telemedicine, telehealth, e-health and m-Health). Satisfaction is conceptualised as the state of enormously complex human thoughts embodied in any format of an evaluation of the targeted phenomenon. Oliver [64] defines satisfaction as ‘…the summary psychological state resulting when the emotion surrounding disconfirmed expectations is coupled with the consumer’s prior feelings about the consumption experience’. According to Tse and Wilton [65] satisfaction refers to ‘the consumer’s response to the evaluation of the perceived discrepancy between prior expectations (or some other norm of performance) and the actual performance of the product as perceived after its consumption’. A study by Yi [66] defined that satisfaction is ‘an evaluation rendered that the (consumption) experience was at least as good as it was supposed to be’. Within the IS context, satisfaction comprises consumers’ in-depth perception of pleasurable accomplishment of service, and the loyalty of commitment to the service provider [67]. For example, consumers compare their perception of product or service performance based on their expectations and some norms of performance [66] which contributes to the formation of satisfaction judgements.

**Fig 1:**
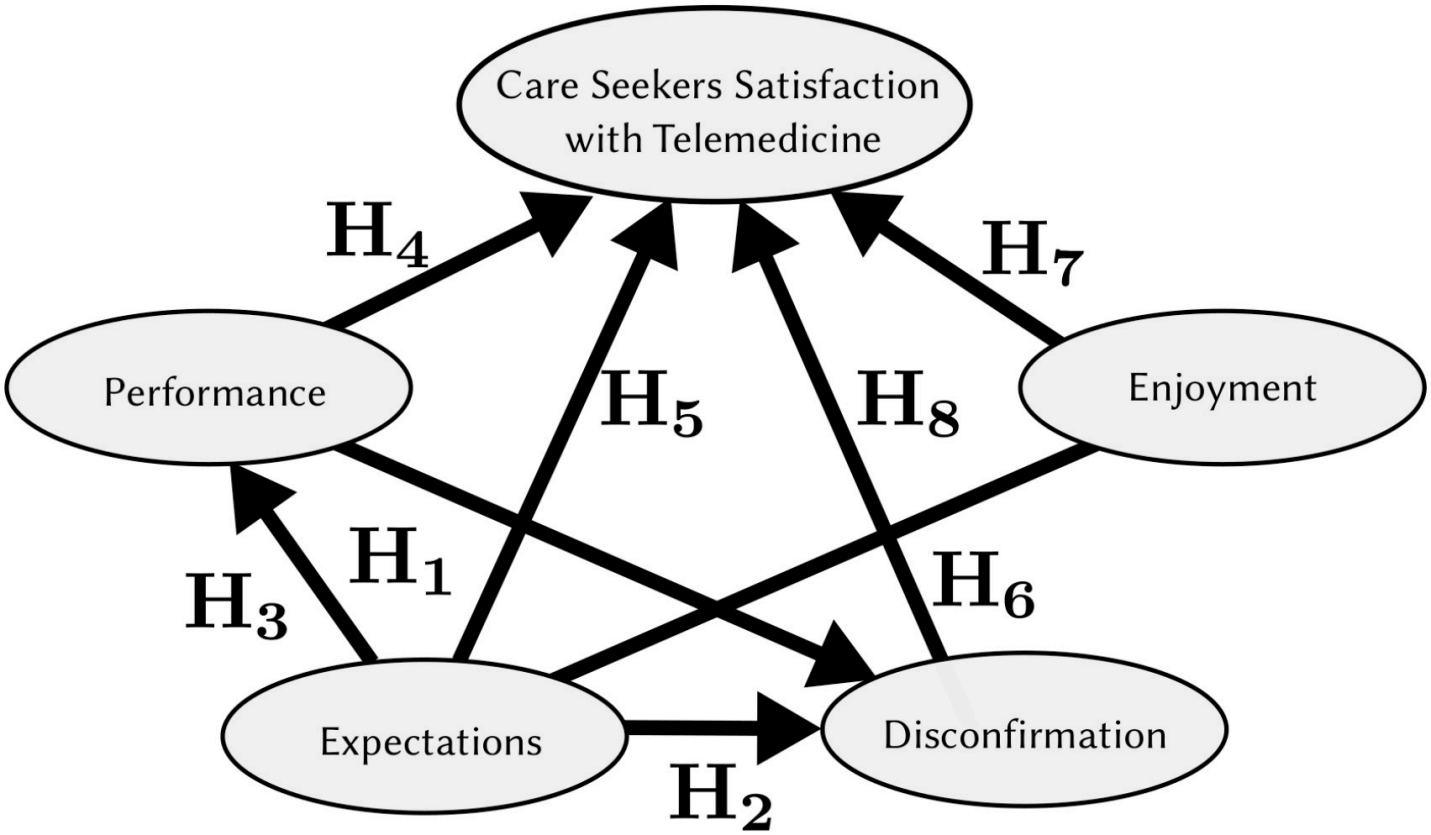
Research model.

Linking EDT, and SCT with care seeker’s satisfaction judgements will provide convincingly an informative way to explore how these antecedents interact and combine these perceptions with standards levels to form their satisfaction judgements [68]. Further, it may expand the generability and applicability of the EDT [69] and SCT frameworks [48]. We argue that EDT and SCT are justifiable because simplified EDT models are often unable to explain more complex phenomena, whereas extended framewoks can [63]. Further, this proposed research model (see Fig 1) identifies the associations between cognitive determinants (i.e., expectations, performance and disconfirmation) along with an affective (i.e., enjoyment) determinant contribute to satisfaction judgement [70]. Motivated by prior research on the EDT and SCT, and analysis of the proposed model postulating four antecednts to satisfaction, the following hypotheses are posited to investigate the research question.

### Expectations

Expectations refer to ‘a set of pre-exposer beliefs about the product’ [41] or service. Expectations as ‘subjective notions of things to come or a type of hypothesis formulated by the consumer’ [57]. Within the IS context, Lankton and Wilson [48] defined that expectations are perceptions of forthcoming service performance that are commonly thought to reflect what an individual believes or anticipates. For example, within e-services, expectations are predictions related to information and service quality [48]. Another study by Zeithaml, Berry, and Parasuraman [71] confirmed that external communications to consumer and service quality information contribute to the formation of expectations. Oliver and Bearden [72] found that disconfirmation (i.e., the fulfilment of expectations) of expectations dominantly influence customer evaluation and judgement of product/service performance.

To measure assimilation effects on EDT research, Lankton et al. [69] noted that when assimilation effects occur; expectations and disconfirmation will have a positive impact on satisfaction. This indicates that an individual finds the differences between initial expectations and actual performance small enough to rely on their initial expectations when forming satisfaction judgements [69]. The links between disconfirmation and satisfaction represent a contrast effect, indicating that an individual finds initial expectations and performance significant enough that he/she relies on discrepancy during satisfaction evaluation [69]. Within telemedicine context, for instance, if a patient finds a positive discrepancy (i.e., performance is better than initial expectations), he/she feels happy and contented than if the discrepancy is negative (i.e., performance is worse than expected) [69]. Consistent with these findings, the following hypotheses are proposed:

- **Hypothesis 1** (**H**_1_): Expectations positively influence patients’ satisfaction in telemedicine usage.
- **Hypothesis 2** (**H**_2_): Expectations positively influences disconfirmation relating to telemedicine usage.
- **Hypothesis 3** (**H**_3_): Expectations positively influences performance relating to telemedicine usage.

### Performance

Performance in this study refers to an individual’s (i.e., patient) post-consumption perception (i.e., belief) about how the technology (i.e., telemedicine) performed on his/her pre-consumption expectation during the use period [63]. Performance refers to the extent of an individual’s expressive dimension of phycological preference [66]. For example, consumers compare (i.e., disconfirmation) their perception of product or service performance with a set of standards (i.e., expectations or some norm of performance) and perceived performance primarily functions for disconfirmation [66]. Within the service context, Halstead, Hartman, and Schmidt [73] revealed that performance and disconfirmation are conclusively related and are potential antecedents of consumers’ satisfaction. Within the e-Health adoption context, Lankton and Wilson [48] found positive relationships of performance and expectations with satisfaction.

Lankton et al. [69] confirmed that expectations help to provide a judgement about performance, while performance plays two roles. For example, performance positively influences disconfirmation holding expectations constant, indicating the higher performance perceived by an individual, the more likely performance will exceed his/her expectations, resulting in positive disconfirmation [69]. Also, performance plays a mediating role between expectations and satisfaction which can reflect the assimilation effect of performance on expectations [69]. Within telemedicine context for example, if a patient perceives the discrepancy between telemedicine service expectations and performance is small enough; performance should have a substantial mediating effect on his/her satisfaction judgement [69]. Consistent with prior studies, the following hypotheses are proposed:

- **Hypothesis 4** (**H**_4_): Performance positively influences patients’ satisfaction in telemedicine usage.
- **Hypothesis 5** (**H**_5_): Performance positively influences disconfirmation relating to telemedicine usage.

### Disconfirmation

Disconfirmation occupies a central position in satisfaction research as a crucial intervening factor arises from discrepancies between prior expectations, and actual performance that generates satisfaction and dissatisfaction judgements [61]. Spreng, MacKenzie, and Olshavsky [74] revealed that disconfirmation judgements suggest that feelings of satisfaction arise when consumers (i.e., patients) compare their perceptions of the performance of services according to their expectations. Disconfirmation in the present study refers to the cognitive comparison between a patient’s pre-consumption service expectations and post-consumption telemedicine service performance. Halstead et al. [73] found that consumers comparison standards (i.e., expectations), disconfirmation beliefs, service performance, quality, affective responses, attribution, and equity judgements, are dominant sources and predictors of satisfaction judgement. Another study by Anderson and Sullivan [15] acknowledged that disconfirmation is a dominant antecedent of satisfaction. Consistent with prior studies, the following hypothesis is proposed:

- **Hypothesis 6** (**H**_6_): Disconfirmation positively influences patients’ satisfaction in telemedicine usage.

### Enjoyment

The integration of individuals perceptual, evaluative, and psychological cognitions generate their consumption satisfaction [66]. Evidence suggests that the enjoyment construct was extracted from Social Cognitive Theory (i.e., form of affect) [48]. According to SCT, enjoyment or attitude represents an individual’s affective reactions towards his/her behaviour and feelings of pleasantness/unpleasantness [48]. Consistent with Lankton and Wilson [48], enjoyment in this study refers to the perception that the use of telemedicine services will be enjoyable/pleasant in its own right. ‘Enjoyment is characterised by a sense of novelty or accomplishment’ [75]. Within technology adoption, Trevino and Webster [76] reminded that if an activity ‘feels good’ and is intrinsically motivating, individuals are more likely involved in that activity for its own sake, while Venkatesh [38] defined that ‘the pleasure and inherent satisfaction derived from a specific activity’. Within e-health, Lankton and Wilson [48] for instance, asserted that if individuals perceive the services enjoyable it will increase their expectations of service performance, while Yi [66] noted that both expectations and performance contribute to forming their satisfaction judgements. Consistent with these findings, the following hypotheses are proposed:

- **Hypothesis 7** (**H**_7_): Enjoyment positively influences patients’ satisfaction in telemedicine usage.
- **Hypothesis 8** (**H**_8_): Enjoyment positively influences expectations in telemedicine usage.

## Research methodology

This research has drawn from a large project, which predominantly explores barriers, facilitators and expectations of telemedicine adoption in rural public hospitals settings in Bangladesh. This study was shaped in quantitative analysis and divided into two sections. The first section includes comprehensive literature review to develop study’s conceptual model. The second section aims to evaluate the questionnaire, empirically validate the proposed research model and hypotheses using a two-staged PLS-SEM and DNN approach. SmartPLS v.3.2.7 was used for data analysis. We believe that applying DNN will be useful in recognizing both linear and non-linear relationships [43] related to normality, linearity, and homoscedasticity [77].

Literature indicates that neural networks learns complex relationships among variables to provide solutions to difficult problems; and consistently reliable in terms of accuracy of findings [78]. Tan et al. [77], revealed that ANN is estimated to be a more robust method for providing with higher prediction accuracy and outperforming other conventional regression analyses. However, due to its ‘black box’ operational nature it is inappropriate for validating hypothesis of casual relationships [77, 79] thus; PLS-SEM approach fills the position.

Within the interoperability and explainability context, this study employed three different explanation methods to validate the estimation of the variables’ contributions in the “black box” network model [80]. The interpretability methods used include Shapley Additive exPlanations (SHAP) [81], Local Interpretable Model-Agnostic Explanations (LIME) [82] and Gradients [83]. Literature indicates that Explainability methods often vary in performance according to domains of application. For example, Lundberg and Lee [81] revealed that the Shapley method is persistently appropriate to better interpret the predictions of complex models in various tasks while, Atanasova et al. [84] showed that gradient-based explanations perform best for natural language processing tasks. LIME and SHAP have shown better performance as general-purpose model explanation methods [81, 82]. We believe that applying the three methods will be advantageous in obtaining a better estimation of variable importance and predictive accuracy in our model. These methods are essential to interpret a prediction model [81] correctly, and they compute an estimation of the contribution of each feature for the predictions [85]. This research utilises the Python programming language along with the Python Standard Library consisting of NumPy, Keras, Sklearn, and Pandas to develop the proposed Neural Network model. Further, this study uses the TensorFlow library, SHAP, LIME, and GradientTape to implement the interpretability and explainability methods.

### Instrument development

To test the research model and hypotheses, a questionnaire was developed, and constructs of the conceptual model were assigned [10]. Primarily, a conceptual model with key constructs, corresponding indicators, and scales was synthesised from existing, established research (see S1 Appendix) [86], and extended/adapted to fit the proposed study [87, 88]. It was then further refined through data collected from participant observation, group discussions, and in-depth interviews before finalising the constructs, indicators, and scales, leading to the establishment of a survey instrument to collect data to validate the hypotheses and the model empirically [89]. A seven-point Likert scale was utilised (i.e., 1 = very strongly disagree to 7 = very strongly agree) [48], which persisted appropriate scale for healthcare surveys [90]. A group of management information systems professionals examined the survey questionnaire for logical consistency, contextual relevance, terminology and measurement content clarity [91]. A pre-test was conducted to examine the wording, sequence, length and format of questionnaire items [92]. A group of fifteen Bangladeshi PhD students were invited to pre-test the questionnaire due to their academic skills, research experience and knowledge regarding Bangladesh telemedicine services. Using their feedback, an iterative rectification process was performed until the questionnaire reached an acceptable range of criterion validity [10]. The questionnaire was initially developed in English, translated into the local language (Bangla), retranslated into English and adjusted so that both versions were comparable to ensure internal validity [93]. The survey questionnaires were distributed with written consent forms to respondents who agreed to participate in the survey. The consent forms reinforced that participation was voluntary and they could withdraw their involvement anytime. The respondents returned the completed questionnaires and the signed consent forms.

### Sample and data collection

The survey process commenced after receiving written approval from the Directorate General of Health Services (DGHS), Ministry of Health and Family Welfare (MOHFW) of Bangladesh and ethical clearance from Research Ethics and Integrity authority at Griffith University, Australia. A cross-sectional survey of telemedicine users was conducted in 2017 in three Upazilas (sub-districts) telemedicine centres in Bangladesh. For this study, 500 rural patients who received telemedicine services at least once from any selected telemedicine centres in the past 12 months constitute the sampling frame. The study excluded non-users because patients in Bangladesh cannot access to telemedicine services without a physician’s referral. Non-telemedicine users including those unable to communicate effectively in any form were excluded from the study. Comprehensive data collection procedures have been described previously [10]. The sample was drawn from selected telemedicine centres using a multistage random sampling design. At first, three districts—Pabna, Khulna and Satkhira where telemedicine services are available were selected randomly. From these three districts, three Upazila telemedicine centres—Bera, Dacope and Devhata were randomly selected as survey implementation site. From the patient lists collected from the selected telemedicine centres, 500 users were randomly selected, consisting of proportionate samples from Bera (*n* = 53), Dacope (*n* = 242), and Devhata (*n* = 205) Upazila telemedicine centres. Each group was statistically representative of the telemedicine population with commonalities in telemedicine infrastructure and clinical methods provided by the government. It is imperative to note that telemedicine in public hospitals has not yet been adopted in every part of the country.

Patients’ addresses and phone numbers were collected from the selected telemedicine centres. Eligible individuals were contacted by phone and invited to participate in face-to-face interviews at a telemedicine centre during office hours. Those unable to travel to the centres were asked to participate according to their convenience. A closed-form interviewer-administered questionnaire was used because several questions were relatively technical and would be hard for respondents with limited literacy to interpret on their own. The study met the sampling target by obtaining 500 valid responses that were scrutinised for completeness. Eight samples were excluded due to incomplete responses; 492 samples were preserved for analysis. The survey’s demographics are presented in Table 1.

**Table 1:** Demographic characteristics of the sample.

Measure	Items	Frequency	Percentage(%)
Gender	Male	206	41.9
	Female	286	58.1
Age	≥ 18 and ≤ 20	64	13.0
	≥ 21 and ≤ 30	158	32.1
	≥ 31 and ≤ 40	106	21.5
	≥ 41 and ≤ 50	88	17.9
	≥ 51	76	15.4
Education	Illiterate	68	13.8
	Primary	104	21.0
	Secondary	178	36.2
	Higher secondary	64	13.0
	Bachelor	51	10.4
	Masters and above	27	5.5

Source: Zobair et al. [10].

## Data analysis and results

### Measurement model

The measurement model was evaluated by testing internal consistency reliability, indicator reliability, convergent validity, and discriminant validity [94]. Except for DISC 4 (0.680), all standardised outer loadings for each indicator in the model (see Table 2) were higher than the threshold value of 0.70, confirming indicator reliability [94, 95]. The outer loadings of indicator DISC 4 (0.680) are close to the threshold value of 0.70 and are confirmed acceptable. The indicators’ outer loadings between 0.40 to 0.70 should only be considered for removal if this increases composite reliability and AVE above the threshold value [94]. Both Cronbach’s alpha and composite reliability (> 0.70) (see Table 2) confirmed the model’s statistical significance and established strong evidence of internal consistency reliability [94]. The Cronbach’s alpha for ENJ (0.67) is less than the threshold value of (> 0.70) but is considered acceptable in exploratory research ranging from 0.60 to 0.70 [94]. Convergent validity (≥ 0.50) was evaluated using the average variance extracted (AVE) values for each construct (see Table 2) [94]. AVE values were higher than > 0.50, demonstrating high levels of convergent validity [96]. The AVE values for each construct explain the variance of more than half of their corresponding indicators, validating convergent validity [95, 97].

**Table 2:** Measurement model assessment.

Latent Constructs	Indicators	Stand. Loading	AVE	Comp. Reliability	Cronbach’s Alpha	R^2^	AdjustedR^2^
Expectations	EXP1	0.820	0.691	0.870	0.777	0.437	0.436
	EXP2	0.830					
	EXP3	0.845					
Performance	PERF1	0.830	0.628	0.834	0.705	0.281	0.279
	PERF2	0.815					
	PERF3	0.728					
Disconfirmation	DISC1	0.805	0.554	0.832	0.732	0.518	0.516
	DISC2	0.783					
	DISC3	0.701					
	DISC4	0.680					
Enjoyment	ENJ1	0.796	0.604	0.821	0.673		
	ENJ2	0.750					
	ENJ3	0.785					
Satisfaction	SAT1	0.725	0.577	0.845	0.755	0.535	0.531
	SAT2	0.748					
	SAT3	0.792					
	SAT4	0.772					

Note. EXP = expectations; PERF = performance; ENJ = enjoyment; DISC = disconfirmation; SAT = satisfaction; Stand. = Standardised; Comp. = Composite.

Discriminant validity defines the extent to which a construct in a model is distinct from other constructs by empirical standards [97]. A construct accepts more variance from its assigned items than from any other constructs [97]. The findings (in bold) in the correlation matrix (see Table 3) confirm that the square root of the AVE for each construct is higher than the correlation with other constructs confirming acceptable discriminant validity [94].

**Table 3:** Fornell-larcker criterion for discriminant validity coefficients.

	**DISC**	**ENJ**	**EXP**	**PERF**	**SAT**
DISC	**0.744**				
ENJ	0.659	**0.777**			
EXP	0.680	0.661	**0.831**		
PERF	0.560	0.577	0.530	**0.792**	
SAT	0.620	0.610	0.669	0.541	0.759

Note. The square root of AVE in bold. EXP = expectations; PERF = performance; ENJ = enjoyment; DISC = disconfirmation; SAT = satisfaction

Additionally, this study measured the distinctiveness of a latent construct using the heterotrait-monotrait ratio (HTMT) criterion test. Literature indicates that the HTMT criterion is essential to evaluate the constructs’ discriminant validity [98–100]. The cut-off scores should be smaller than 0.85 (more strict threshold) or 0.90 (more lenient threshold) to interpret the results [98, 99]. The results (see Table 4) demonstrate that HTMT is significantly less than 0.85 or 0.90, authenticating that all measured constructs illustrated their discriminant validity [98] except two pairs. Our results provided evidence that we established discriminant validity among all constructs. However, we cannot confirm discriminant validity between two pairs of constructs such as ENJ and DISC, and EXP and ENJ contain the HTMT value of 0.925 and 0.910, respectively, suggesting a lack of discriminant validity.

**Table 4:** Heterotrait-monotrait ratio (HTMT) for discriminant validity coefficients.

	**DISC**	**ENJ**	**EXP**	**PERF**	**SAT**
DISC					
ENJ	0.925				
EXP	0.883	0.910			
PERF	0.770	0.829	0.708		
SAT	0.820	0.851	0.872	0.724	

Note. EXP = expectations; PERF = performance; ENJ = enjoyment; DISC = disconfirmation; SAT = satisfaction

Further, the bootstrapping technique was applied for testing whether the HTMT value is significantly less than 1 [94]. Our results demonstrated that HTMT is considerably less than 1 [98]. We found that neither of the confidence intervals includes the value 1 [94] except ENJ and DISC, EXP and ENJ, similar to the previous test. This indicates that the lower and upper bounds of the confidence interval of HTMT for the relationships between ENJ and DISC are 0.925 and 1.007, EXP and ENJ are 0.910 and 1.007, thus below the threshold value of 0.90 or significantly smaller than 1 (95% percentile confidence interval), suggesting a lack of discriminant validity [94]. We could not establish discriminant validity between these constructs in HTMT test. These results are unexpected and need further investigation.

### Structural model

**Fig 2:**
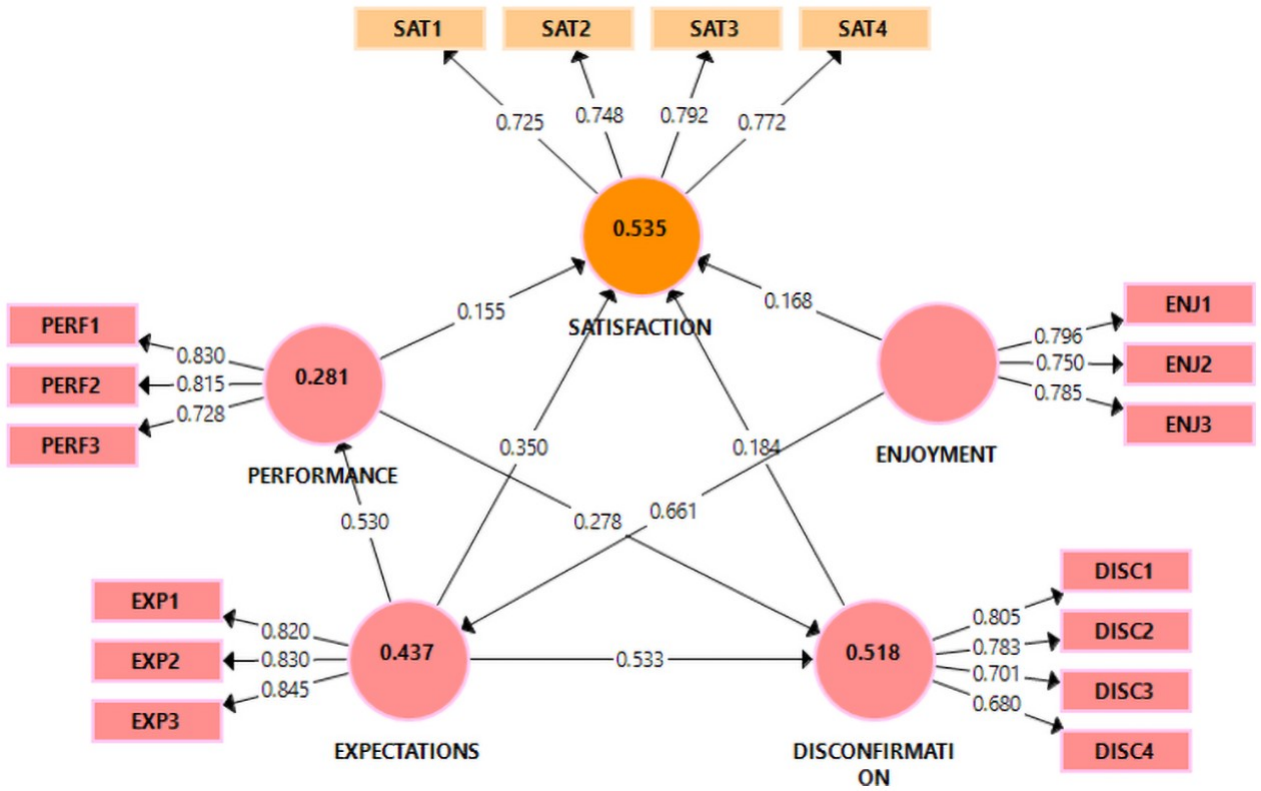
Final PLS-SEM structural model forecasting satisfaction with telemedicine.

The structural model confirmed that five hypotheses were validated by the relationships (*p* < 0.01) (see Fig 2). The four proposed latent constructs had significant effects (see Tables 2 and 5) on patients’ satisfaction (SAT) related to telemedicine usage and adoption. The findings demonstrate that the relationships between expectations (EXP) and satisfaction (SAT) (*β* = 0.350, *t* = 6.089, *p* < 0.01), performance (PERF) and satisfaction (SAT) (*β* = 0.155, *t* = 2.833, *p* < 0.01), disconfirmation (DISC) and satisfaction (SAT) (*β* = 0.184, *t* = 3.773, *p* < 0.01) and enjoyment (ENJ) and satisfaction (SAT) (*β* = 0.168, *t* = 3.030, *p* < 0.01) were statistically significant, confirming support for *H*_1_, *H*_4_, *H*_6_ and *H*_7_ (see Table 5). An *R*^2^ ≈ 0.53 indicates that about 53% of the variance (i.e., SAT) in the model was jointly explained by the EXP, PERF, DISC and ENJ constructs. The results were between moderate and substantial *R*^2^ values (i.e., 33%, 67%) [97], suggesting a high predictive capability of this satisfaction model. Further, *R*^2^ demonstrates each construct’s significance and its associative contribution to overall *R*^2^ [101].

**Table 5:** Structural model assessment.

	Hypotheses	Path Coefficient (*β*)	SE	*t*	*p*	*f* ^2^	Decision
H1	EXP→SAT	0.350***	0.058	6.089	0.000	0.118	Supported
H2	EXP→DISC	0.533***	0.045	11.756	0.000	0.423	Supported
H3	EXP→PERF	0.530***	0.038	14.086	0.000	0.390	Supported
H4	PERF→SAT	0.155***	0.055	2.833	0.005	0.031	Supported
H5	PERF→DISC	0.278***	0.045	6.201	0.000	0.115	Supported
H6	DISC→SAT	0.184***	0.049	3.773	0.000	0.032	Supported
H7	ENJ→SAT	0.168***	0.055	3.030	0.002	0.027	Supported
H8	ENJ→EXP	0.661***	0.031	21.016	0.000	0.777	Supported

Note.

*p < 0.10;

** *p* < 0.05;

*** *p* < 0.01 (two-tailed) confidence intervals for significance testing.

EXP = expectations; PERF = performance; ENJ = enjoyment; DISC = disconfirmation; SAT = satisfaction.

The structural model further revealed that four other hypotheses were statistically significant and validated. The relationships between expectations (EXP) and disconfirmation (DISC) (*β* = 0.533, *t* = 11.756, *p* < 0.01), expectations (EXP) and performance (PERF) (*β* = 0.530, *t* = 14.086, *p* < 0.01), performance (PERF) and disconfirmation (DISC) (*β* = 0.278, *t* = 6.201, *p* < 0.01), and enjoyment (ENJ) and expectations (EXP) (*β* = 0.661, *t* = 21.016, *p* < 0.01), were statistically significant. This validates *H*_2_, *H*_3_, *H*_5_, and *H*_8_ (see Table 5) demonstrating that these constructs have a substantial influence on their endogenous latent constructs.

The practical relevance of significant effects should be investigated by testing the effect sizes of the latent constructs’ relationships [98]. Literature indicates that the *f*^2^ values ranging from 0.020 to 0.150, 0.150 to 0.350, or larger or equal to 0.350, demonstrating weak, medium or large effect size, respectively [98]. The *f*^2^ value (see Table 5) for all hypothesised relationships ranges from 0.027 to 0.777 (weak to large) in our sample. The effect size is a measure of the magnitude of an effect independent of sample size, and it is unusual and unlikely that most constructs will have a large effect size in the model [98].

Additionally, the present study assessed overall saturated model fit evaluation. Due to recent PLS-SEM developments, the overall model fit can be estimated using standardised root mean squared residual (SRMR), squared Euclidean distance, and the geodesic distance [99]. The measure of fit (SRMR) and the test of overall model fit squared Euclidian distance (*d*_*ULS*_) and the geodesic distance (*d*_*G*_) is preferable in casual research [98, 99]. The discrepancy between the two matrices is measured by squared *d*_*ULS*_, *d*_*G*_, and, the SRMR [98, 99] assuming, at a 5% significance level [98]. The recommended threshold value of SRMR should be below 0.080, and all discrepancy measures (*d*_*ULS*_ and *d*_*G*_) should be below the 95% quantile of their reference distribution (*HI*_95_), indicating that the estimated model was not rejected at a 5% significance level [98]. In our model (see Table 6), all values of discrepancy were below the 95% quantile of their corresponding reference distribution (*HI*_95_) except (*d*_*ULS*_) authenticating acceptable overall model fit. Further, the SRMR is below the threshold value of 0.080, indicating a good model fit [98]. This test has recently been developed in PLS-SEM and IS researchers are encouraged to use this evaluation in casual research [98, 99].

**Table 6:** Overall saturated model fit evaluation.

Discrepancy	Value	HI_95_	Decision
SRMR	0.071	0.055	Supported
*d*_*ULS*_	0.767	0.465	Supported
*d*_*G*_	0.273	0.220	Supported

### Deep neural network analysis

Machine learning enables to develop any complex model to obtain new knowledge through an iterative process of learning with structured data pre-processed from big data [43]. Deep learning is a machine learning approach attracting much attention from academic and industrial communities [102]. It has been widely used in many theoretical and practical fields of investigations [103] including computer vision, natural language processing [104], image recognition, language translations [105] and health trends and predictions [43]. The DNN comprise an information processing structure consisting of multiple layers and interconnected neuron units with fully functional relationships [106].

**Fig 3:**
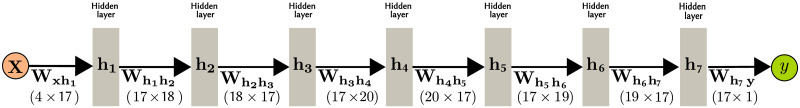
Deep Neural Network model forecasting satisfaction with telemedicine. *W*_*ij*_ represents the parameter matrix consisting of weights from layer *i* to layer *j*. The dimension of each weight matrix is give in ().

The configuration of our proposed DNN model (see the network model in Fig 3 consists of one input layer **X** with four input variables namely, performance (*X*_1_), expectations (*X*_2_), disconfirmation (*X*_3_), and enjoyment (*X*_4_), seven hidden layers (*h*_1_ − *h*_7_) with seventeen (*N* = 17), eighteen (*N* = 18), seventeen (*N* = 17), twenty (*N* = 20), seventeen (*N* = 17), nineteen (*N* = 19), and seventeen (*N* = 17) neurons respectively. Neural network dynamics are set in motion when neurons in the first hidden layer receive inputs (as constant values in tensors or arrays) from the input layer. The input values are multiplied with corresponding weights and added to the bias to generate intermediate values. The intermediate values are then transformed using a non-linear activation function [107]. The outputs of the activations from a layer serve as the inputs for the next layer. The process of intermediate value and activation computations are performed at each layer until the output layer [78]. The central part of DNN functionality is the learning and training procedure in which the errors determined at the output layer are successively reduced by adjusting the weights and biases throughout the network [108]. Through iterative weights adjustment, the network learns the best set of weights to estimate the target variable, in our case satisfaction [43]. The principle of adjusting weights is to reduce the errors and optimise the classification outcomes of the network model [109].

One of the central challenges in DNN model development is the best model architecture identification and selections. Model selection is the process of selecting the hyperparameters of the best-performing model. The hyperparameters must be set manually after finding the optimal hyperparameter configuration as per the model selection process [110].

The present study determined the hyperparameters of the model using a random search strategy (using the Keras-tuner library). The random search strategy is used to seek out the optimal neural network model [111]. We designed the random search consisting of eight runs with randomly assigning 4 to 20 neurons to each layer. Each run included 250 trials in random order (see the search results in Tables 8 to 15). The detailed hyperparameter search space is shown in Table 7. In each run, we gradually incremented the number of hidden layers until we obsereve no decrement in loss (see Table 15). Literature indicates that the random search method has recently become a popular alternative to grid search [111]. Choosing a random search for this research is a more efficient method than a grid search. Typically, only a subset of a model’s tuneable hyperparameters is vital for optimising performance [111]. Thus, we chose a random search technique for the hyperparameters optimisation using three runs, 250 experiments each with 1000 epochs.

**Table 7:** Hyperparameter search space.

Hyperparameter	Run 1	Run 2	Run 3	Run 4	Run 5	Run 6	Run 7	Run 8
Number of hidden layers	1	1, 2	1 to 3	1 to 4	1 to 5	1 to 6	1 to 7	1 to 8
Number of neurons	4 to 20	4 to 20	4 to 20	4 to 20	4 to 20	4 to 20	4 to 20	4 to 20
Activation functions	tanh, ReLU, PReLU, LeakyReLU, ELU							

**Table 8:** Hyperparameter search results summary (Run 1). Top 5 Models out of 250 candidate models from Run 1.

	Model 1		Model 2		Model 3		Model 4		Model 5	
	HL	N	HL	N	HL	N	HL	N	HL	N
Number of neurons	1	19	1	18	1	13	1	9	1	10
Activation	PReLU		PReLU		PReLU		tanh		tanh	
RMSE	0.12244		0.12550		0.12793		0.12827		0.12829	

Note. HL = Hidden Layer; N = Number of neurons.

**Table 9:** Hyperparameter search results summary (Run 2). Top 5 Models out of 250 candidate models from Run 2.

	Model 1		Model 2		Model 3		Model 4		Model 5	
	HL	N	HL	N	HL	N	HL	N	HL	N
Number of neurons	1	14	1	7	1	18	1	16	1	12
	2	12	2	16	2	10	2	8	2	11
Activation	PReLU		PReLU		tanh		tanh		tanh	
RMSE	0.10873		0.11907		0.12097		0.12414		0.12436	

Note. HL = Hidden Layer; N = Number of neurons.

**Table 10:** Hyperparameter search results summary (Run 3). Top 5 Models out of 250 candidate models from Run 3.

	Model 1		Model 2		Model 3		Model 4		Model 5	
	HL	N	HL	N	HL	N	HL	N	HL	N
Number of neurons	1	17	1	19	1	13	1	17	1	10
	2	19	2	13	2	12	2	12	2	12
	3	17	3	20	3	13	3	9	3	8
Activation	tanh		tanh		PReLU		PReLU		tanh	
RMSE	0.10294		0.10649		0.10852		0.11214		0.11554	

Note. HL = Hidden Layer; N = Number of neurons.

**Table 11:** Hyperparameter search results summary (Run 4). Top 5 Models out of 250 candidate models from Run 4.

	Model 1		Model 2		Model 3		Model 4		Model 5	
	HL	N	HL	N	HL	N	HL	N	HL	N
Number of neurons	1	18	1	19	1	19	1	18	1	14
	2	19	2	15	2	14	2	17	2	9
	3	4	3	6	3	13	3	14	3	12
	4	10	4	6	4	14	4	7	4	17
Activation	tanh		PReLU		tanh		tanh		PReLU	
RMSE	0.09732		0.10080		0.10466		0.10610		0.10677	

Note. HL = Hidden Layer; N = Number of neurons.

**Table 12:** Hyperparameter search results summary (Run 5). Top 5 Models out of 250 candidate models from Run 5.

	Model 1		Model 2		Model 3		Model 4		Model 5	
	HL	N	HL	N	HL	N	HL	N	HL	N
Number of neurons	1	14	1	19	1	17	1	15	1	16
	2	14	2	14	2	12	2	6	2	11
	3	16	3	13	3	17	3	16	3	18
	4	17	4	15	4	19	4	4	4	13
	5	6	5	7	5	9	5	17	5	20
Activation	tanh		tanh		tanh		tanh		tanh	
RMSE	0.09801		0.09913		0.10197		0.10375		0.10620	

Note. HL = Hidden Layer; N = Number of neurons.

**Table 13:** Hyperparameter search results summary (Run 6). Top 5 Models out of 250 candidate models from Run 6.

	Model 1		Model 2		Model 3		Model 4		Model 5	
	HL	N	HL	N	HL	N	HL	N	HL	N
Number of neurons	1	7	1	10	1	17	1	14	1	11
	2	16	2	16	2	12	2	8	2	9
	3	10	3	18	3	17	3	11	3	4
	4	17	4	9	4	19	4	11	4	7
	5	11	5	9	5	9	5	15	5	17
	6	9	6	16	6	9	6	11	6	18
Activation	tanh		tanh		tanh		tanh		tanh	
RMSE	0.09509		0.09820		0.10610		0.10375		0.10892	

Note. HL = Hidden Layer; N = Number of neurons.

**Table 14:** Hyperparameter search results summary (Run 7). Top 5 Models out of 250 candidate models from Run 7.

	Model 1		Model 2		Model 3		Model 4		Model 5	
	HL	N	HL	N	HL	N	HL	N	HL	N
Number of neurons	1	17	1	18	1	17	1	20	1	12
	2	18	2	9	2	16	2	9	2	10
	3	17	3	9	3	13	3	16	3	18
	4	20	4	19	4	8	4	8	4	5
	5	17	5	6	5	7	5	19	5	7
	6	19	6	5	6	17	6	18	6	10
	7	17	7	18	7	13	7	20	7	11
Activation	tanh		tanh		tanh		tanh		tanh	
RMSE	0.07719		0.08734		0.08783		0.09711		0.09879	

Note. HL = Hidden Layer; N = Number of neurons.

**Table 15:** Hyperparameter search results summary (Run 8). Top 5 Models out of 250 candidate models from Run 8.

	Model 1		Model 2		Model 3		Model 4		Model 5	
	HL	N	HL	N	HL	N	HL	N	HL	N
Number of neurons	1	14	1	18	1	16	1	14	1	19
	2	13	2	14	2	14	2	18	2	15
	3	12	3	18	3	16	3	11	3	17
	4	18	4	10	4	15	4	5	4	11
	5	19	5	6	5	12	5	5	5	16
	6	10	6	11	6	15	6	4	6	18
	7	9	7	18	7	15	7	7	7	15
	8	18	8	7	8	8	8	11	8	14
Activation	tanh		tanh		tanh		tanh		tanh	
RMSE	0.08800		0.08883		0.08893		0.08969		0.09110	

Note. HL = Hidden Layer; N = Number of neurons.

The RMSE scores obtained by the top five models, out of 250 trials in each Run, are shown in Tables 8 to 15. We observe that Model 1 in Run 7 is the best-performing model. It is worth noting that this study tested several activation functions, including ReLU, PReLU, LeakyReLU, ELU, and tanh. Literature indicates that these activation functions possess advantages and disadvantages [112]. However, we found that the tanh activation function outperforms others in our experiments. Literature indicates that tanh is a commonly used activation function and leads to faster convergence during training [113].

The Hyperparameter search results of Run 7 are exhibited in Table 14 where the RMSE scores obtained by the top 5 models are 0.07719, 0.08734, 0.08783, 0.09711, and 0.09879 respectively, showing that Model 1 is the best-performing model.

Our findings demonstrated minimal or no differences between train and test errors (see Table 16), confirming a non-overfitting and non-underfitting model [108]. The neural network’s efficiency and prediction accuracy is measured by the root mean square error (RMSE) [114]. The findings (see Table 16) confirmed that the values of RMSE with minimal training and testing errors (see Table 16), indicating an optimal fit DNN model.

**Table 16:** RMSE per epoch for the optimised DNN model.

Epoch	Train error	Test error
25/1000	0.0795	0.0757
115/1000	0.0657	0.0653
193/1000	0.0652	0.0664
263/1000	0.0597	0.0594
306/1000	0.0594	0.0596
426/1000	0.0591	0.0591
507/1000	0.0587	0.0595
603/1000	0.0585	0.0591
795/1000	0.0578	0.0587
831/1000	0.0576	0.0581
985/1000	0.0567	0.0573

This study employed SHAP which is a game-theoretic approach that uses shapely values [85]. Shapely values express the average contributions of each player in a team to the outcome of a game, in our case contributions of each feature to the prediction. LIME builds a linear regression model from perturbed samples similar to a test instance and assigns contribution scores to the features using a LASSO regularisation method [82]. Gradient (of a model with respect to the input variables) draws a measure of variation which attributes the degrees of changes of a prediction to the input variables [80]. In the above methods, a higher score indicates the corresponding variables is more important than the variables with lower scores.

Note that these are local interpretation methods, meaning that the explanations provided are for a single instance. In the following, $\varphi_{j}^{\left(i\right)}\in R$ represents the local importance of the *j*th variable for the *i*th instance and $I_{j}\in R$ represents the global importance of the *j*th variable. In SHAP *I*_*j*_ is computed by summing the absolute shapely values across the instances [85] which is given by:

$$\begin{array}{c} I_{j}=\sum_{i}^{n}\left|\varphi_{j}^{\left(i\right)}\right| \end{array}$$

where, *n* is the total number of instances. LIME provides global importance by a module named SP-LIME [82] where the importance of the *j*th variable is computed as:

$$\begin{array}{c} I_{j}=\sqrt{\sum_{i}^{n}\left|\varphi_{j}^{\left(i\right)}\right|} \end{array}$$

In gradient based method, the global importance *I*_*j*_ is computed as [80, 83]:

$$\begin{array}{c} I_{j}=\frac{1}{n}\sum_{i}^{n}\left|\varphi_{j}^{\left(i\right)}\right| \end{array}$$

Here, $\varphi_{j}^{\left(i\right)}$ is the gradient of a model *f*(**X**) with respect of the *j*th variable. We obtain the gradient $\frac{\partial f(X)}{\partial x_{j}^{\left(i\right)}}$ using GradientTape.

### SHAP, LIME, and Gradient-based interpretability assessment

The eXplainable Artificial Intelligence (XAI) methods have gained popularity in both academia and industries to interpret internal logic and the outcome of “black-box models” [115]. A plethora of current literature indicates that various interoperability methods exist in machine learning; however, it is often unclear how these approaches are related and preferable over another [81]. These approaches differ in feature importance measures. From a functional point of view, LIME fits approximate model predictions locally [116]; SHAP measures the entire class of additive feature attributions both locally and globally [81]; Gradients based explainability methods link features importance variability by measuring the change in gradients of the outputs with respect to the inputs [116]. Due to the functional variability, the results of LIME, SHAP and Gradient-based methods can vary. Slack et al. [117] have shown that LIME is more unstable than SHAP due to the random input perturbations used by LIME to construct the training set for explanation extraction. Likewise, Lai et al. [116] observed variations in the outputs of SHAP and LIME methods.

**Fig 4:**
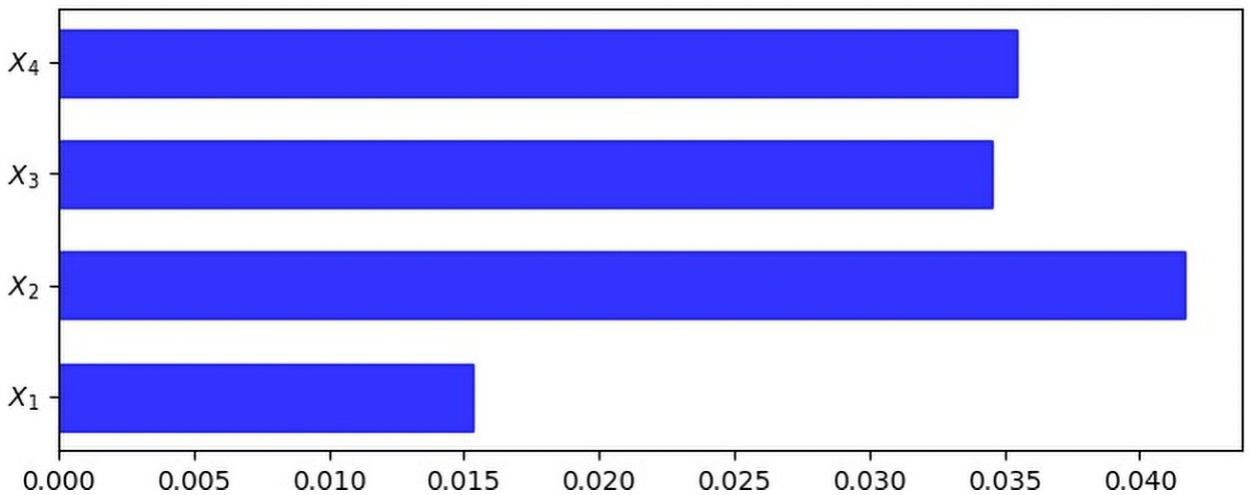
SHAP values to estimate variables’ prediction contributions. Note. *X*_1_ = performance; *X*_2_ = expectations; *X*_3_ = disconfirmation; *X*_4_ = enjoyment.

**Fig 5:**
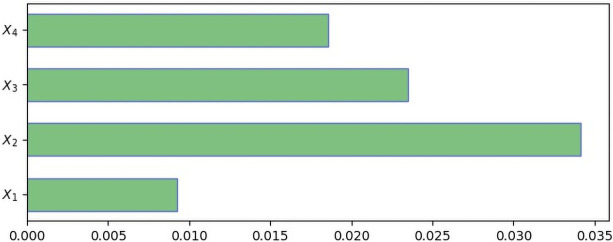
LIME values to estimate variables’ prediction contributions. Note. *X*_1_ = performance; *X*_2_ = expectations; *X*_3_ = disconfirmation; *X*_4_ = enjoyment.

**Fig 6:**
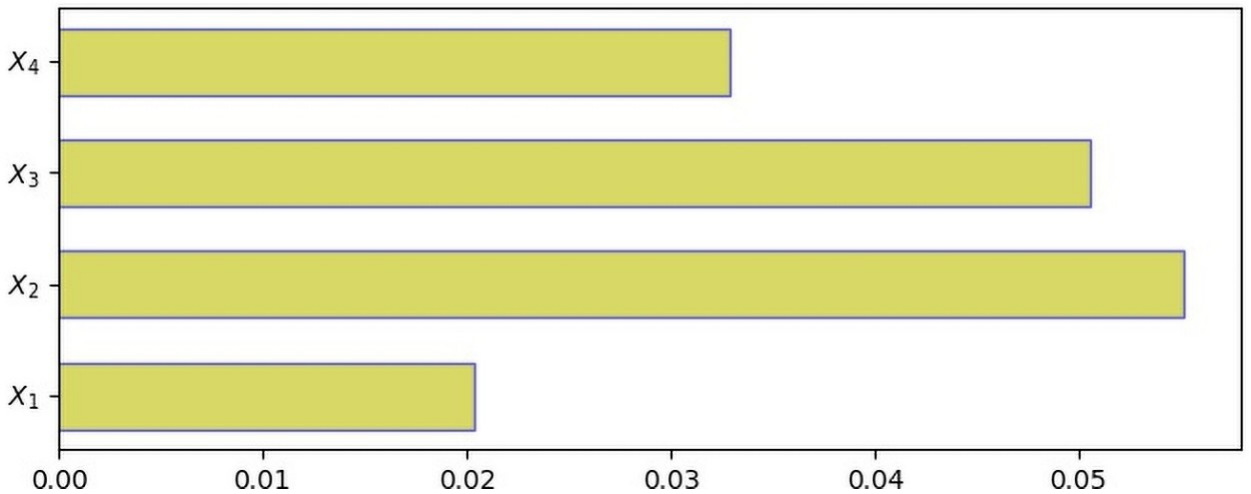
Gradient values to estimate variables’ prediction contributions. Note. *X*_1_ = performance; *X*_2_ = expectations; *X*_3_ = disconfirmation; *X*_4_ = enjoyment.

Given the emphasis on the interpretability and explainability of the Machine Learning models, specifically DNNs, this study experiments with comprehensive interpretations using SHAP, LIME, and Gradient-based methods. The magnitudes of the outputs of the methods associated with each feature represent how significant the feature’s contribution is to the prediction of a model [115]. The outcomes of SHAP, LIME, and Gradient-based methods on our study are presented in Figs 4–6, respectively. The greater the magnitude, the more significant its contribution to the model’s prediction [115].

The findings from the SHAP, LIME and GradientTape analyses (see Figs 4–6) show that *X*_2_ (i.e., expectations) is the most dominant contributor, while *X*_3_ (i.e., disconfirmation), *X*_4_ (i.e., enjoyment) and *X*_1_ (i.e., performance) are second, third and fourth-order contributors respectively. SHAP produces slightly different orders for *X*_4_ and *X*_3_ (however, the magnitudes of *X*_3_ and *X*_4_ are very close). Except for the scarcely ordered variability in one instance, all three SHAP, LIME, and GradientTape methods are consistent on the features’ contributions to predicting care seekers’ satisfaction with telemedicine experience in rural Bangladesh settings. Nonetheless, our findings still imply that each construct (i.e., performance, expectations, disconfirmation and enjoyment) is a strong determinant regardless of high, medium, or low magnitudes, predicting health seekers’ satisfaction with telemedicine services in rural Bangladesh.

## Discussion of the results

The empirical findings from DNN and PLS-SEM analyses, authenticated that four antecedents—expectations, performance, disconfirmation, and enjoyment—play vital roles in forming, predicting and forecasting care seekers’ satisfaction with telemedicine, laying the groundwork for further policy intervention and research.

Based on the DNN model the findings provide evidence that expectations had the most significant effect on satisfaction making it the strongest predictor of care seekers’ satisfaction with telemedicine. This model illustrates that higher levels of patients’ satisfaction are reported in efforts to rationalise their earlier high expectations associated with telemedicine services [53]. This indicates that expectations remain crucial for assessing and estimating patients’ satisfaction levels. In keeping with the findings from PLS-SEM we have confirmed that expectation is the dominant predictor of satisfaction with telemedicine. This verifies Bearden and Teel [53] observations, that expectations are presumed to accomplish the function of an adaptation level in the way they defined the standard (i.e., expectations) against which subsequent performance is being judged. Our results corroborated by Bhattacherjee [62] revealed that expectation is a dominant antecedent contributing to the process of satisfaction formation. Applied to telemedicine adoption success, our findings tie in with a recent study by Zobair et al. [10] and acknowledged that expectations could be compared with a baseline of patient satisfaction with telemedicine experience. Bhattacherjee [62] found satisfaction is a dominant predictor of their usage continuance. The research findings are consistent with prior studies [44, 62, 69, 118], validating that expectations is a leading antecedent contributed to forming and predicting satisfaction with telemedicine in rural hospital settings in Bangladesh.

The findings from DNN analysis provide evidence that disconfirmation is the second highest antecedent predicting satisfaction which is congruent with the PLS-SEM analysis. The fact that both approaches are statistically significant, provide evidence that disconfirmation has dominating effects on satisfaction with telemedicine. The research findings are congruent with Lankton et al. [42] observations, that individuals compare their initial expectations with perceived service performance fostering satisfaction judgement, while Spreng and Page Jr [36] for example, claimed that disconfirmation is a distinct cognitive state that is subjectively perceived by an individual which can be measured independently. Further, our findings validate Ho and Wu [119] remarks, that positive disconfirmation has a substantial effect on satisfaction in using the Internet, while Lankton and McKnight [63] recognised that positive disconfirmation (i.e., usefulness and ease of use) influences consumers’ satisfaction judgement. This indicates that pshchological dimension of satisfaction provides a means that wheather an individual’s expectations are confirmed or disconfirmed by their perceived performance [44]. Consistant with this view, this study argues that care seekers optimise the discrepancy between their perceived pre-consumption expectations and post-consumption performance to control their satisfaction judgement pertinent to telemedicine. These imply that patients’ satisfaction is distinct from their cognitive disconfirmation process [73] which in turn affect their satisfaction judgements related to telemedicine experience. From these research findings, we conclude that disconfirmation is a strong antecedent that played a key role to predict patients’ satisfaction with telemedicine.

From a hedonic point of view, the findings from DNN and PLS-SEM analyses provide strong evidence that enjoyment has a significant impact on satisfaction (third highest position in both PLS-SEM and DNN analyses), making it another dominant predictor of patients’ satisfaction with telemedicine. This implies that patients’ satisfaction judgement is formed by their positive perceptions and enjoyment concerning high service quality and reliability. This is particularly relevant to telemedicine service processes consisting of several attributes (i.e., video conferencing, health providers attitude, long waiting) which may be perceived enjoyable or unenjoyable turnings into patients’ satisfaction or dissatisfaction. The research findings are largely congruent with Venkatesh [120] observations, who found strong associations between perceived pleasure and satisfaction towards performing the behaviour in technology adoption. This study argues that enjoyment plays a vital role in the formation of satisfaction indicating that patients’ enjoyment raises, the levels of satisfaction also increase concurrently. Our findings appear rational and expected since we argue that patients’ emotions and feelings strongly affect the level of their cognitive influence on satisfaction judgement. A similar pattern of findings by Davis, Bagozzi, and Warshaw [121] voiced that enjoyment has a dominant effect on technology usage intentions. These findings support Carlsmith and Aronson [122] remarks, that dissonance (psychological discomfort) influences individuals’ hedonic levels related to their satisfaction judgement. Our findings show the evidence that enjoyment is a leading antecedent to predicting patients’ satisfaction with telemedicine in rural hospital settings in Bangladesh.

Findings from PLS-SEM and DNN analyses reveal that performance is the fourth contributor to forming patients’ satisfaction with telemedicine. The DNN model has comparative advantages to interpret the prediction of complex systems better and is congruent with Tan et al. [77] statements that DNN model is estimated to be a more robust method for providing higher prediction accuracy and outperforming other conventional regression analyses. Both the DNN and PLS-SEM approaches confirm that performance has a significant impact on satisfaction authenticating its statistical significance. Our findings support Serrano et al. [40] statement that service performance strongly influences patients’ satisfaction, while Lankton and Wilson [48] found that performance has a dominating effect on patients’ satisfaction judgement. Similar findings by Lankton and McKnight [63] echoed that service performance positively influences satisfaction and has the most significant impact on continuous intentions. We argue that if telemedicine consistently delivers high-quality services (i.e., performance) it is more likely to have satisfied care seekers resulting in the continuance of usage. A similar conclusion highlighted by Garcia and Adelakun [45] acknowledged that convenience (i.e., performance) and appropriate telemedicine service profoundly influence patients’ satisfaction. These findings are largely consistent with prior studies [24, 29, 36, 48, 66, 73, 123], confirming that performance is a dominant antecedent to patients’ satisfaction with telemedicine.

Our findings authenticated that four antecedents—expectations, performance, disconfirmation, and enjoyment—play vital roles in forming, predicting, and forecasting care seekers’ satisfaction with telemedicine. They differ from other satisfaction studies within the telemedicine literature. Existing literature [7, 17, 45, 124] investigated satisfaction in the context of telemedicine but did not (surprisingly) apply EDT theory to explore how consumers’ expectations, performance, and disconfirmation contribute to their satisfaction judgments. They point to research on the antecedents to satisfaction with telemedicine, without validating satisfaction using EDT, which remains a leading theory to study satisfaction proposed by Oliver [23, 24]. This implies that telemedicine’s existing satisfaction literature is not theoretically up to date in the way the investigations had been approached, particularly in emerging economies’ context. Against this backdrop, the study’s findings fill these significant gaps contributing to health informatics and behavioural literature by clarifying the complex interplay between patients’ satisfaction and determinants of continuity behaviour in telemedicine’s domain.

From a methodological point of view, some researchers theorised that satisfaction within telemedicine research had methodological and analytical limitations. For example [22], applied Technology Acceptance Model (TAM) to investigate health providers satisfaction with telemedicine, while Serrano et al. [40] applied EDT to identify patient satisfaction with telemedicine for diabetic retinopathy screenings. Interestingly, they adopted a linear modelling approach. Venkatesh and Goyal [41] suggested that linear models are unable to reveal complexities that are expected in theories and lead to oversimplifying the complexity of the combined effects of the components. Following their work, this research applies a unique non-linear methodological approach using Deep Neural Network (DNN), recognising both linear and non-linear relationships [125].

Additionally, this research used the PLS-SEM method, a full-fledged variance-based approach ideal for linear, non-linear, recursive, and non-recursive structural models [98]. The present research investigates how DNN, and PLS-SEM can assist telemedicine in enabling predictions better, increasing patient recovery and satisfaction, and supporting health professionals in better quality decision-making. Hence, our findings confirm that the four constructs (i.e., expectations, performance, disconfirmation, and enjoyment) significantly affect predicting patient satisfaction decisions and subsequent future continuity with telemedicine in rural settings. Failure to recognise these determinants and impacts on health seekers’ satisfaction with telemedicine will undervalue attempts at implementation in emerging economies.

## Contributions and managerial implications

The theoretical and methodological contributions of this research illustrate the importance of customised theorisation of existing EDT and SCT along with the introduction of unique Machine Learning and Artificial Intelligence technology to answer the crucial research questions by better grounding the phenomenon of satisfaction with telemedicine. This raises satisfaction’s contributory benchmark to uncover how its antecedents contribute to predicting patients’ decisions towards telemedicine’s future continuity. Second, the apparent strength of this research is the large sample size and the interpretations of a range of key contributors that play a vital role in strengthening and widening telemedicine scope in developing countries’ rural settings. Third, this research explores the complex interplay between satisfaction and its antecedents illuminating novel insights into predictions of patients’ future telemedicine usage trends thereby assisting health professionals, academics, policymakers, and IS community to higher quality informed decisions for people-centred future models of care. Fourth, this research complements a body of new knowledge related to patients’ unique behavioural characteristics towards their satisfaction judgements and delivered additional insights into effective decisions strategies to pursuit successful telemedicine deployment in large scale. Fifth, this research employs a two-staged DNN and PLS-SEM approach to delineate the elements of satisfaction that persistently contribute to imparting newfound knowledge and best practices in both developed and developing countries contexts, validating a methodological contribution. Sixth, the inclusion of three interoperability and explainability methods (SHAP, LIME and Gradient) has made a significant contribution to promoting accurate interpretation and better estimation of the factors/variables’ contributions and predictions accuracy in the network model. Additionally, this research opens a new avenue for Machine Learning and Artificial Intelligence-based health informatics research and provides valuable directions to academic and IS practitioners to integrate approaches to strengthen telemedicine sustainability.

From a managerial standpoint, this research explores distinctive patients’ cognitive and affective nature within the healthcare environment that has theoretically been linked to satisfaction [126]. The findings from this research illustrate that care seekers’ expectations are presumed to accomplish the function of adaptation to new conditions/services in the way they defined the standard against which subsequent performance is being judged, while high service performance positively impacts on their continuance intentions. This suggests that health providers should strive to provide high-quality services leading to satisfied patients resulting in their retention. Further, health providers need to prioritise patients’ coordination, simplistic referral process to specialised physicians, prompt service delivery, and supportiveness that lead to patients positive impression (i.e., disconfirmation) towards future service continuity. This implies that the psychological dimension of satisfaction mediates whether an individual’s expectations are confirmed or disconfirmed by their perceived performance. This research proves that enjoyment plays a vital role in the formation of satisfaction, indicating that as patients’ enjoyment increases, the levels of satisfaction also increase. Given these findings, the health providers, and policymakers should design effective strategies, develop favourable policy guidelines and implement satisfaction management plans for achieving goals.

From a practical point of view, these findings have important implications for information systems, particularly in health informatics research. The discoveries associated with theory and practices complement each other well in concept generation and development of telemedicine healthcare systems in both developed and developing countries rural settings. Our proposed model emerged as a dominant benchmark model, played a strong role in forming, predicting and forecasting patients’ satisfaction with telemedicine and similar ICT facilitated healthcare systems such as e-Health, telehealth, and m-Health. Furthermore, several issues pertaining to the intricate patterns of antecedents to patients’ satisfaction with telemedicine were deliberated, to shed new lights on fostering the deployment of this provision in emerging economies. Additionally, the validity, robustness and functionality of this robust model has been confirmed, signifying implications across health industries to fostering its effective deployment in Bangladesh and globally. However, it is apparent that more in-depth investigations further required in this context.

### Limitations and future research directions

Nevertheless, some limitations do exist in this study and should be considered when interpreting findings. First, this study reflected only on the prediction of care seekers’ satisfaction with telemedicine. Future research should examine the health providers’ satisfaction with telemedicine, this may provide additional evidence. Second, only public telemedicine systems were included in the current study. Private and NGO provided telemedicine systems could be involved in future research to observe the variances between public and private service interests. Third, this study focused on a few antecedents to care seekers’ satisfaction with telemedicine. Future research could be broadened by including age, gender, education, economic conditions, health literacy and situational effects that may significantly influence patients’ satisfaction related to telemedicine provision. Fifth, data for this study were collected in 2017, demonstrating a significant limitation. The time-series data can be collected for future research that may provide additional evidence related to telemedicine satisfaction. Finally, the present study was limited to Bangladesh. Combining this study with cross-sectional data from similar settings would provide extensive understandings of patients’ satisfaction with telemedicine in a global context.

## Conclusion

This research successfully verified and determined the potential antecedents that play key roles in predicting and forecasting patients’ satisfaction with telemedicine experience in rural public hospitals in Bangladesh. The apparent strengths of this research are to effectively introduce artificial neural network along with PLS-SEM to explore the elements of intricate patterns of patients’ satisfaction judgements with telemedicine experience, in order to boosting the ability of effective organisational decision making to respond more rapidly to future healthcare problems. The integration of EDT and SCT has promoted a complete theoretical view of satisfaction formation process in telemedicine framework. Our study, which validates that expectation and performance are predictors of disconfirmation, while disconfirmation accurately captures an individual’s judgement of their perceived discrepancy between expectations and performance [36]. Congruent with the findings from Davis et al. [121], we observed that enjoyment dominantly contributes to forming patients’ satisfaction decision towards telemedicine usage. These findings underscore the importance of how cognitive factors (i.e., EDT) and emotional (i.e., affective) factor (i.e., SCT) remarkably play critical roles in the formation of satisfaction. This indicates that abandoning enjoyment (i.e., factor) to understand the antecedent to satisfaction with telemedicine would be irrational and undesirable, but complementing this notion would be extremely fruitful [127]. This indicates that enjoyment is a new component of EDT, demonstrating a contributory benchmark to explore the antecedents to satisfaction with telemedicine. However, due to theoretical and practical limitations, the present research did not test additional determinants might be involved in patients’ cognitive evaluations [54] in the arena of satisfaction with telemedicine. Finally, this study recommends that health providers, policymakers, and stakeholders may set realistic and achievable goals in building telemedicine into an institutionalised health infrastructure for providing better quality patient care with greater flexibility through modernised, specialised healthcare support for medically underprivileged rural communities in Bangladesh and similar settings. Rural and remote communities often have limited access to dedicated public medical facilities and most lack private after-hours medical practitioners. Hence, these sparsely populated regions should continue to be a priority.

**Research highlights**

- A theoretically sustained prediction model contributed to health informatics research.
- Deep Neural Network and PLS-SEM advance Expectation Disconfirmation Theory and Social Cognitive Theory adaptability.
- Four antecedents of satisfaction were discovered in telemedicine settings.
- Machine Learning and Artificial Intelligence technologies forecasting antecedents to satisfaction with telemedicine.

## Supporting information

S1 Appendix Summary of constructs with measurement items. (PDF)

S1 Table Demographics characteristics of the sample and survey responses. (PDF)
