# Supplementary material for: Forecasting care seekers satisfaction with telemedicine using machine learning and structural equation modeling
Source: PLoS One. 2021 Sep 24;16(9):e0257300. doi: 10.1371/journal.pone.0257300 (PMC8462681; doi:10.1371/journal.pone.0257300)
Supplement: S1 Appendix — (PDF) [file pone.0257300.s001.pdf]

Forecasting Care Seekers Satisfaction With Telemedicine Using Machine Learning and Structural Equation Modeling

Khondker Mohammad Zobair, Louis Sanzogni, Luke Houghton, Md. Zahidul Islam

S1 Appendix. Summary of constructs with measurement items.

Table 1: **Summary of constructs with measurement items.**

**Constructs**	**Indicators**	**Statements**	**Sources**
Expectations	EXP1	I expect that telemedicine will provide me with timely responses from specialised physicians.	[1, 2, 3, 4, 5]
	EXP2	I expect that telemedicine will enhance communication between my physicians and me.	
	EXP3	I expect that telemedicine will offer me better healthcare access.	
Performance	PERF1	Telemedicine provides me with timely responses to all my health needs.	[1, 2, 3, 4, 1]
	PERF2	Telemedicine provides me with better access to healthcare services.	
	PERF3	Telemedicine provides me with information relevant to my health needs.	
Disconfirmation	DISC1	Telemedicine supports my health needs much better than I expected.	[2, 3, 4]
	DISC2	Telemedicine physicians understand my health problems much better than I expected.	
	DISC3	Telemedicine completes all tasks well and much better than I expected.	
	DISC4	My overall experience with telemedicine is much better than I expected.	
Enjoyment	ENJ1	I enjoy using telemedicine services that are provided by my hospital.	[1, 6, 5]
	ENJ2	I find that using telemedicine is enjoyable for me.	
	ENJ3	I believe that the actual process of using telemedicine via videoconferencing is pleasant for me.	
Satisfaction	SAT1	Telemedicine services in my hospital have satisfied my needs.	[1, 5, 2, 3, 4]
	SAT2	Telemedicine satisfied me with timely responses from specialised hospitals.	
	SAT3	Telemedicine satisfied me with the communication between my specialist physician and me.	
	SAT4	I have been satisfied with the telemedicine services that performed well for the first time.	

References

[1] Lankton NK, Wilson EV. Factors influencing expectations of e-health services within a direct-effects model of user satisfaction. E-Service Journal. 2007;5(2):85–112.

[2] Lankton NK, McKnight HD. Examining two expectation disconfirmation theory models: assimilation and asymmetry effects. Journal of the Association for Information Systems. 2012;13(2):1.

[3] Lankton N, McKnight DH, Thatcher JB. Incorporating trust-in-technology into Expectation Disconfirmation Theory. The Journal of Strategic Information Systems. 2014;23(2):128–145.

[4] Spreng RA, Page Jr TJ. A test of alternative measures of disconfirmation. Decision Sciences. 2003;34(1):31–62.

[5] Zobair KM, Sanzogni L, Sandhu K. Expectations of telemedicine health service adoption in rural Bangladesh. Social Science & Medicine. 2019;238:112485.

[6] Venkatesh V. Determinants of perceived ease of use: Integrating control, intrinsic motivation, and emotion into the technology acceptance model. Information systems research. 2000;11(4):342–365.
