# Supplementary material for: Forecasting care seekers satisfaction with telemedicine using machine learning and structural equation modeling
Source: PLoS One. 2021 Sep 24;16(9):e0257300. doi: 10.1371/journal.pone.0257300 (PMC8462681; doi:10.1371/journal.pone.0257300)
Supplement: S1 Table — (PDF) [file pone.0257300.s002.pdf]

Forecasting Care Seekers Satisfaction With Telemedicine Using Machine Learning and Structural Equation Modeling

Khondker Mohammad Zobair, Louis Sanzogni, Luke Houghton, Md. Zahidul Islam

S1 Table. Demographics characteristics of the sample and survey responses.

The survey of telemedicine users was conducted from care seekers in three sub-district telemedicine centres in Bangladesh. Comprehensive data collection procedures have been previously published in [1]. The survey's demographics are presented in Table 1.

Table 1: **Demographic characteristics of the sample**

Measure	Items	Frequency	Percentage(%)
Gender	Male	206	41.9
	Female	286	58.1
Age	≥ 18 and ≤ 20	64	13.0
	≥ 21 and ≤ 30	158	32.1
	≥ 31 and ≤ 40	106	21.5
	≥ 41 and ≤ 50	88	17.9
	≥ 51	76	15.4
Education	Illiterate	68	13.8
	Primary	104	21.0
	Secondary	178	36.2
	Higher secondary	64	13.0
	Bachelor	51	10.4
	Masters and above	27	5.5

Source: Zobair et al. [1].

We collected 500 responses and scrutinised the responses for completeness. Eight samples were excluded due to incompleteness. The complete 492 responses are given in Table 2.

Table 2: **Survey responses**

ID	sat	perf	exp	disc	enj	ID	sat	perf	exp	disc	enj
1	28	21	21	28	21	51	28	21	21	28	21
2	28	21	21	28	21	52	26	18	18	28	21
3	25	18	17	23	18	53	26	18	18	28	21
4	27	17	18	21	17	54	28	21	21	28	21
5	28	21	21	28	21	55	28	21	21	28	21
6	26	20	19	27	18	56	27	19	21	26	19
7	28	21	21	28	21	57	28	21	21	28	21
8	28	21	21	28	21	58	28	21	21	28	21
9	26	18	21	21	16	59	28	21	21	28	21
10	24	18	16	22	15	60	28	21	21	28	21

Continued on next page

Table 2 – continued from previous page

ID	sat	perf	exp	disc	enj	ID	sat	perf	exp	disc	enj
11	21	18	15	23	18	61	23	17	16	23	18
12	21	15	17	20	15	62	27	17	20	26	17
13	22	17	15	20	15	63	28	21	21	28	21
14	26	21	18	28	21	64	28	21	21	28	21
15	27	19	21	27	20	65	28	19	21	27	19
16	28	21	21	28	21	66	28	21	17	24	19
17	28	21	21	28	21	67	28	21	21	28	21
18	28	21	19	24	19	68	28	21	21	27	21
19	28	21	21	28	15	69	28	20	20	26	20
20	28	21	19	24	21	70	27	21	21	28	21
21	26	17	19	28	17	71	26	16	21	26	18
22	25	17	19	26	17	72	26	17	21	28	21
23	25	19	19	26	17	73	26	19	21	28	21
24	22	19	19	25	19	74	28	21	21	28	21
25	26	21	20	26	20	75	28	21	21	28	21
26	27	19	20	26	20	76	28	21	21	28	21
27	26	21	20	26	20	77	28	21	21	26	18
28	28	21	20	26	21	78	28	21	21	28	21
29	26	20	20	26	20	79	28	19	21	28	21
30	28	21	21	28	21	80	28	20	21	26	21
31	26	20	19	26	19	81	27	19	20	26	19
32	26	20	19	27	18	82	25	19	19	27	17
33	26	20	19	27	17	83	26	18	21	28	20
34	26	20	19	27	18	84	28	21	21	28	21
35	27	20	19	27	18	85	28	21	21	28	21
36	26	20	19	27	18	86	28	21	21	28	21
37	26	20	19	26	18	87	28	21	21	28	21
38	26	18	17	21	15	88	27	19	20	26	19
39	28	21	21	28	21	89	26	20	19	26	17
40	28	21	21	28	21	90	28	19	20	28	19
41	28	21	21	28	21	91	27	21	21	27	21
42	28	21	21	28	21	92	28	21	20	27	21
43	28	21	21	28	21	93	28	17	20	27	18
44	28	21	21	28	21	94	26	15	20	25	16
45	28	20	21	28	21	95	26	20	20	25	21
46	28	21	21	28	21	96	27	21	21	25	19
47	28	21	21	28	21	97	26	19	20	24	19
48	28	21	21	28	21	98	26	19	18	23	15
49	28	21	21	28	21	99	27	21	21	28	21
50	28	21	21	28	21	100	28	21	21	28	21
101	28	21	21	28	21	151	28	21	21	28	21
102	28	21	21	28	21	152	28	21	21	28	21
103	27	21	21	26	18	153	28	21	21	28	21
104	28	21	21	28	21	154	28	21	21	28	21
105	28	21	21	28	21	155	21	18	16	22	15
106	28	21	18	28	21	156	22	15	17	21	16
107	28	21	21	28	21	157	24	19	16	21	17
108	28	21	21	28	21	158	21	17	16	22	17
109	26	19	20	21	17	159	23	18	17	22	17
110	22	18	16	24	18	160	22	17	16	23	19
111	22	18	16	24	18	161	24	18	17	23	18
112	24	19	15	23	18	162	28	21	21	28	17
113	20	19	17	24	19	163	22	16	17	21	17
114	22	19	15	23	19	164	28	21	21	26	21

Continued on next page

Table 2 – continued from previous page

ID	sat	perf	exp	disc	enj	ID	sat	perf	exp	disc	enj
115	21	19	15	23	17	165	25	16	19	22	19
116	22	18	15	21	18	166	24	18	17	21	16
117	28	21	21	28	21	167	27	17	19	24	20
118	28	21	21	28	21	168	25	20	17	24	19
119	26	18	21	21	16	169	27	19	18	24	16
120	21	18	16	23	18	170	25	19	17	23	16
121	21	18	16	23	18	171	24	19	17	23	18
122	20	18	15	22	16	172	25	16	19	23	18
123	21	18	16	22	18	173	28	21	21	28	21
124	21	18	15	23	16	174	24	16	17	23	20
125	18	20	15	22	16	175	27	21	21	28	21
126	22	16	15	23	13	176	28	19	21	27	18
127	22	18	15	24	17	177	28	21	21	24	21
128	28	19	20	28	19	178	27	18	19	25	18
129	28	21	17	26	15	179	28	18	19	25	20
130	27	20	20	26	21	180	22	21	18	24	18
131	23	21	18	22	18	181	27	20	20	27	18
132	27	21	18	26	20	182	28	17	19	22	21
133	24	18	21	22	16	183	27	18	18	25	16
134	25	21	19	28	17	184	28	20	20	25	20
135	28	21	21	28	18	185	28	20	19	23	21
136	28	21	21	28	21	186	28	19	21	28	20
137	28	21	21	27	21	187	27	20	17	24	19
138	28	19	21	28	19	188	25	20	18	24	20
139	28	21	21	28	21	189	24	17	20	26	19
140	28	21	21	28	21	190	25	20	18	22	19
141	28	21	21	28	21	191	28	21	21	26	21
142	28	21	21	28	21	192	25	18	20	25	17
143	28	21	21	28	21	193	26	19	20	26	20
144	28	21	21	27	21	194	28	20	21	28	20
145	28	21	21	28	21	195	28	21	21	28	15
146	28	21	21	22	18	196	25	18	18	24	18
147	28	21	20	27	21	197	28	20	21	26	21
148	28	21	21	28	21	198	28	21	20	27	21
149	28	21	21	28	21	199	28	21	21	24	21
150	28	21	21	27	20	200	27	21	18	26	21
201	28	21	21	28	21	251	26	19	20	24	19
202	28	21	21	28	21	252	27	20	21	25	18
203	27	21	21	25	21	253	22	18	21	27	18
204	27	19	17	21	16	254	22	21	21	26	21
205	28	20	20	26	20	255	28	19	21	25	21
206	27	18	18	25	18	256	27	20	21	27	20
207	24	19	18	25	18	257	26	17	21	28	21
208	28	21	21	28	19	258	26	20	20	27	20
209	20	15	15	20	15	259	28	17	21	28	21
210	24	20	20	26	16	260	26	21	20	25	20
211	21	18	15	20	15	261	24	18	18	23	18
212	28	21	21	24	21	262	26	16	17	23	18
213	25	21	19	24	19	263	24	17	16	23	15
214	26	19	21	28	21	264	24	18	18	26	20
215	27	19	18	22	18	265	25	17	16	25	18
216	28	15	21	24	21	266	25	16	19	28	19
217	28	21	21	28	21	267	27	18	19	26	18
218	26	15	15	24	15	268	27	18	19	23	16

Continued on next page

Table 2 – continued from previous page

ID	sat	perf	exp	disc	enj	ID	sat	perf	exp	disc	enj
219	28	21	21	26	21	269	26	21	21	25	21
220	28	21	21	26	21	270	27	18	19	26	18
221	28	21	21	26	21	271	26	18	21	28	21
222	28	21	21	28	21	272	27	20	19	28	19
223	27	18	20	26	21	273	26	18	21	26	18
224	28	21	21	23	21	274	25	20	19	25	17
225	25	20	21	25	18	275	25	20	19	24	18
226	25	20	20	26	20	276	23	20	20	23	18
227	25	20	21	25	18	277	25	19	21	24	19
228	25	20	20	26	18	278	26	18	20	22	20
229	26	17	21	27	21	279	24	17	20	25	18
230	25	20	21	25	18	280	25	19	19	22	20
231	25	20	21	25	18	281	26	20	20	27	19
232	22	20	21	25	18	282	26	20	19	27	20
233	25	20	21	25	18	283	26	20	20	28	19
234	27	19	21	25	21	284	27	17	20	23	19
235	27	19	21	25	21	285	17	20	16	23	15
236	26	21	20	27	19	286	21	17	16	21	16
237	27	19	21	25	21	287	26	19	20	27	20
238	28	21	21	28	21	288	28	21	21	28	21
239	27	19	21	25	21	289	17	20	21	26	21
240	27	19	21	25	21	290	28	21	21	26	21
241	28	20	21	24	21	291	23	18	20	25	19
242	28	19	20	27	20	292	17	12	18	20	15
243	26	18	20	25	19	293	28	21	21	26	21
244	27	19	21	25	21	294	22	17	18	24	20
245	26	20	20	25	17	295	26	17	20	26	19
246	24	18	21	26	18	296	26	17	20	27	19
247	25	17	19	20	17	297	26	18	17	24	18
248	25	18	20	23	18	298	25	18	19	24	18
249	26	19	20	27	18	299	28	18	19	26	15
250	26	17	20	27	19	300	24	18	18	26	18
301	27	18	19	25	19	351	25	21	21	24	17
302	26	16	20	26	18	352	25	21	20	26	19
303	26	20	20	26	19	353	27	18	21	28	21
304	26	20	20	25	19	354	28	21	21	28	21
305	25	18	19	26	19	355	28	21	21	26	21
306	27	20	20	23	19	356	28	21	21	26	21
307	27	18	20	23	19	357	22	19	18	23	19
308	22	20	20	24	18	358	26	18	17	23	16
309	28	20	19	24	19	359	26	21	15	20	15
310	25	20	20	25	20	360	22	18	15	20	15
311	24	18	20	25	20	361	28	21	21	26	21
312	28	21	21	28	21	362	28	21	21	28	21
313	25	21	19	24	19	363	28	21	21	28	21
314	26	17	17	26	19	364	23	15	15	22	15
315	28	21	21	28	21	365	24	18	18	24	18
316	28	20	20	26	19	366	28	21	21	26	21
317	22	21	21	28	21	367	28	21	21	26	21
318	28	21	20	24	21	368	26	20	17	25	19
319	28	21	21	24	21	369	25	21	21	26	21
320	20	15	15	20	15	370	28	21	21	28	21
321	28	20	19	24	21	371	28	20	18	26	21
322	28	20	19	22	21	372	27	19	21	27	21

Continued on next page

Table 2 – continued from previous page

ID	sat	perf	exp	disc	enj	ID	sat	perf	exp	disc	enj
323	25	18	21	22	19	373	23	15	18	23	16
324	24	15	19	24	20	374	23	21	19	27	21
325	28	19	20	28	19	375	22	18	15	20	15
326	25	17	19	24	19	376	27	15	15	20	15
327	25	19	18	25	20	377	28	21	18	26	21
328	28	21	21	26	21	378	22	20	18	25	18
329	28	20	20	28	19	379	21	16	16	22	19
330	25	19	21	24	18	380	22	17	17	26	16
331	28	21	21	24	21	381	28	20	21	26	21
332	26	19	19	25	17	382	28	21	21	26	18
333	28	21	20	28	21	383	27	21	18	27	20
334	28	21	21	28	19	384	28	21	21	26	19
335	28	21	21	28	21	385	28	21	21	28	21
336	28	20	21	28	20	386	28	21	21	28	21
337	28	19	19	24	19	387	28	21	21	28	21
338	27	20	20	27	19	388	27	18	20	26	19
339	23	19	17	23	18	389	27	20	20	28	20
340	28	21	21	26	21	390	27	21	19	26	20
341	26	18	20	25	20	391	27	20	21	27	20
342	28	21	18	28	21	392	28	18	19	26	20
343	28	21	18	28	21	393	28	21	21	28	21
344	25	20	21	28	20	394	28	21	21	28	21
345	28	19	21	27	20	395	28	21	21	28	21
346	28	21	21	28	21	396	28	21	21	28	21
347	28	21	21	28	21	397	20	15	15	21	15
348	26	20	19	26	19	398	27	13	16	23	14
349	26	18	18	26	18	399	22	21	19	25	16
350	28	21	18	26	21	400	26	21	18	22	18
401	28	21	21	28	21	451	28	21	21	26	21
402	28	21	21	28	21	452	28	19	21	28	21
403	28	20	20	28	21	453	25	18	19	26	20
404	28	19	19	28	20	454	26	19	20	26	18
405	27	17	18	26	18	455	27	18	19	27	20
406	28	20	20	27	21	456	28	19	19	26	20
407	27	17	21	28	20	457	27	18	19	23	20
408	28	21	21	26	20	458	25	20	20	28	18
409	27	19	21	27	20	459	28	21	21	28	21
410	28	21	21	28	21	460	28	17	21	27	19
411	27	20	21	27	20	461	28	19	20	28	20
412	27	18	21	27	19	462	26	19	20	28	19
413	27	19	21	25	20	463	28	21	21	28	21
414	27	21	18	27	20	464	28	21	21	28	19
415	28	19	18	27	20	465	28	18	21	27	21
416	27	19	20	26	19	466	28	19	21	27	21
417	26	17	20	26	16	467	28	19	21	28	21
418	26	20	15	28	19	468	28	21	21	28	21
419	26	20	20	25	20	469	28	21	21	28	21
420	28	20	21	26	20	470	28	20	20	28	21
421	28	19	19	25	21	471	27	20	20	26	18
422	28	21	20	27	21	472	28	21	21	28	21
423	28	19	21	27	19	473	28	21	21	28	21
424	26	21	19	27	19	474	28	21	21	28	21
425	27	21	21	27	20	475	28	21	21	28	21
426	28	19	20	26	17	476	27	20	21	25	19

Continued on next page

Table 2 – continued from previous page

ID	sat	perf	exp	disc	enj	ID	sat	perf	exp	disc	enj
427	27	21	21	28	21	477	28	19	21	26	21
428	28	21	21	26	21	478	28	21	19	24	19
429	28	21	17	28	21	479	27	20	20	26	19
430	28	21	21	28	21	480	27	21	20	25	19
431	28	21	21	26	17	481	27	21	21	28	21
432	28	21	21	28	21	482	28	21	21	26	19
433	26	21	21	26	18	483	27	20	21	25	19
434	24	21	15	22	15	484	26	20	21	27	19
435	21	21	18	28	21	485	27	19	21	27	19
436	27	21	21	28	21	486	27	20	20	26	20
437	24	19	18	23	16	487	27	20	20	28	19
438	28	21	21	28	19	488	27	19	21	26	20
439	28	19	17	24	17	489	27	20	20	26	19
440	28	21	21	26	21	490	27	20	19	27	19
441	21	15	15	22	15	491	27	20	20	27	19
442	22	15	15	20	15	492	27	20	20	25	20
443	26	20	19	26	19						
444	22	18	17	24	15						
445	22	17	17	24	16						
446	28	21	21	26	21						
447	26	18	20	26	20						
448	27	18	18	21	19						
449	26	20	19	25	19						
450	28	21	18	28	21						

References

[1] Zobair KM, Sanzogni L, Sandhu K. Expectations of telemedicine health service adoption in rural Bangladesh. Social Science & Medicine. 2019;238:112485.
